# Osseointegrative effect of rhBMP-2 covalently bound on a titan-plasma-spray-surface after modification with chromosulfuric acid in a large animal bone gap-healing model with the Göttingen minipig

**DOI:** 10.1186/s13018-018-0915-x

**Published:** 2018-08-30

**Authors:** Manuel Lingner, Roland Seidling, Lars Johannes Lehmann, Eckhard Mauermann, Udo Obertacke, Markus Ludwig Rupert Schwarz

**Affiliations:** 1 Department for Experimental Orthopaedics and Trauma Surgery, Orthopaedic and Trauma Surgery Centre (OUZ), University Medical Centre Mannheim, Medical Faculty Mannheim, Heidelberg University, Theodor-Kutzer-Ufer 1-3, 68167 Mannheim, Germany; 2 Department for Anaesthesia and Intensive Care Medicine, Asklepios Südpfalzklinik Kandel, Luitpoldstr. 14, 76870 Kandel, Germany; 3 Clinic of Trauma and Hand Surgery, Vincentius-Kliniken gAG Karlsruhe, Südendstr. 32, 76137 Karlsruhe, Germany; 4 Department for Anesthesia, Surgical Intensive Care, Prehospital Emergency Medicine and Pain Therapy, Basel University Hospital, Spitalstrasse 21, 4031 Basel, Switzerland

**Keywords:** BMP-2, Osseointegration, Gap model, Minipig, CSS, TPS, Histomorphometry, Intravital labeling

## Abstract

**Background:**

Bone morphogenetic proteins play an important role as osseointegrative factors. It is used widely in orthopedic research and surgery to enhance the osseointegrative potential of implants, e.g., in spinal fusion or alveolar socket augmentation. The aim of the present study was to investigate the benefit of rhBMP-2 on a titan plasma spray (TPS) layer after a special modification with chromosulfuric acid (CSA) at different postoperative times, regarding osseoconduction and osseoinduction.

**Methods:**

We allocated 27 Göttinger minipigs into three groups consisting of nine animals each. They received four dumbbell-shaped implants in the metaphyseal parts of the femora. The implants had a TPS surface with (CSA group) and without a CSA treatment (TPS group). The former received an additional layer of BMP-2 (BMP-2 group). For the assessment of osseointegration after healing periods of 4, 8, and 12 weeks, histomorphometry was applied to undecalcified specimens after staining according to Masson-Goldner. An intravital labeling with different fluorochromes was used in the gap model. A multivariable analysis with repeated measurement design was performed for statistical evaluation.

**Results:**

We observed several statistical differences in a three-way ANOVA. The comparison between the BMP-2 and the TPS group (two-way ANOVA) showed statistically significant differences in terms of the osseoinduction (osteoid volume), and pronounced for the osseoconduction (bone and osteoid ongrowth), in favor of the BMP-2 group. In the pairwise comparison between BMP-2 and CSA (two-way ANOVA), no statistical significance occurred. The intravital staining with tetracycline, calcein green, and xylenol orange revealed no considerable differences between the groups.

**Conclusion:**

BMP-2, covalently bound on a CSA-treated TPS surface, has positive effects on the osseointegration in the large animal bone gap-healing model over the observation period of 12 weeks.

## Background

Bone morphogenetic proteins play an important role in the proliferation, differentiation, and induction of mesenchymal stem cells as well as in the embryogenesis [1]. BMP-2, currently one of the best-studied bone morphogenetic proteins, is applied increasingly in orthopedic surgery [2]. The working group of Jennissen developed a method for the surface modification which allows a subsequent biocoating by proteins, e.g., BMP-2 [3]. The basis of this principle underlies the production of an ultra-hydrophilic surface on titanium implants with chromosulfuric acid, which leads to a so-called inverse lotus effect, which causes a reduction of the contact angle to < 10° [4]. The procedure has already been published several times in detail and is patented [3, 5–8]. By the addition of anchor molecules, BMP-2 can be bound and retains its biological activity [6, 9]. Thus, the prerequisite for adequate protein binding is the prior treatment with chromosulfuric acid. Previous studies showed that the treatment with chromosulfuric acid alone has an osseointegrative potential [10]. BMPs as differentiation factors are suspected to promote malignant degeneration when used in humans [11].

Gap models have been used since 1980 and earlier in orthopedic research, allowing the investigation of bone repair under special conditions with different questions [10, 12–15].

The animal of our choice, the Göttingen minipig, has been proven to be a reliable animal for examining implants, especially because of the similar characteristics of the mesenchymal stem cells [16], as well as because of a comparable lamellar bone structure [17].

Histomorphometry, as well as the polychrome labeling, finds application in the evaluation of bone remodeling processes [10]. For fluorescence microscopy, in the sense of intravital staining, combinations of different fluorochromes lead to a better resolution of ossification fronts by synergistic effects [18], and this could be shown, e.g., for tetracycline and xylenol orange [19]. For some substances, no spectral analysis is needed, which would require an immense effort for the evaluation [20, 21].

The gold standard in orthopedic research is the histomorphometry, particularly for the assessment of osseointegration, which allows a quantitative assessment on the basis of an established standardized nomenclature since the 1980s [22–24].

The aim of the study was to investigate the osseointegrative force of different titanium surfaces at different postoperative times in respect to a covalently bound layer made of BMP-2 additionally. The application in an animal bone gap-healing model should reveal the potential for osseoinduction and osseoconduction.

## Methods

The animal study was approved by the ethical committee of the Regierungspräsidium Karlsruhe, Abteilung 3 - Landwirtschaft, Ländlicher Raum, Veterinär - und Lebensmittelwesen, Karlsruhe, Germany, under the file no. 35-9185.81/G-27/05.

### Preparation of implants

Cylindrical dumbbell-shaped implants were used with a total length of 30 mm, a gap length of 15 mm, and a resulting gap after implantation with a height of approximately 1.28 mm as reported by Seidling et al. [10]. The surface structure was created on one hand, by the application of titanium plasma spray (TPS group), and on the other hand, by modification with a chromosulfuric acid alone (CSA group) and subsequent covalently bound rhBMP-2 (BMP-2 group). This method was developed and patented by the working group of Jennissen (University of Duisburg-Essen) [3, 7]. The modification is carried out by a treatment with chromosulfuric acid for 60 min at high temperature and subsequent cleaning with EDTA and water [3, 25]. This process leads to an ultra-hydrophilic surface by thickening the oxide layer with improved binding capacity [4]. After this treatment, BMP-2 can be covalently bound stable to the surface and retains its biological activity [25, 26].

The titan implants with the TPS layer in the middle part were produced by the company DOT (Rostock, Germany) and described in more details by Seidling et al. [10]. Modified in the manner described above, they were provided sterile and ready for implantation by the working group of Jennissen (University of Duisburg-Essen) and the company Morphoplant GmbH (Duisburg, Germany).

### Animals and surgery

Twenty-seven Göttinger minipigs (Ellegaard, Dalmose, Denmark) were divided into three groups with nine animals each. In the TPS group, the animals had an average weight of 64.72 kg (SD ± 6.26 kg). In the CSA group, the average weight was 59.5 kg (SD ± 13.85 kg), and in the BMP-2 group, the average weight was 56 kg (SD ± 14.69 kg). The animals were on average 37.7 months old in the mean (SD ± 4.66 months). Thus, the animals were adults, as Christensen had reviewed [27].

We observed a time period of 4, 8, and 12 weeks postoperatively for each group; so, the animals were accordingly divided into three further subgroups.

Each animal received four implants as described previously by Seidling et al. [10]. The implants were implanted on both sides, in the cancellous bone of the distal and proximal part of the femur strictly press-fit and under general anesthesia according to Schwarz et al. [28, 29]. Pre- and postoperative monitoring and housing of the animals were performed in the “Interfakultäre Biomedizinische Forschungseinrichtung (IBF)” of the University of Heidelberg (Germany) up to 14 days postoperatively. They were held for the followed healing period on a farm near Heidelberg until to a few days before the scheduled time point of killing. The animals were fed with “Schweine-Einmastkorn” (17% crude protein) of the company Muskator (Mannheim, Germany). Water was available at any time ad libitum. The animals survived the operation procedure, and there were no perioperative obvious complications observed.

### Intravital staining

For the semiquantitative evaluation of the osseointegration, an intra-vital staining was performed with the substances calcein green (20 mg/kg; Fa. Pfizer, Karlsruhe/Germany), xylenol orange (90 mg/kg; Fa. Waldeck GmbH&CoKG, Münster/Germany), and tetracycline (26 mg/kg; Fa. Waldeck GmbH&CoKG Münster/Germany) [30], according to the scheduled time of application (Table 1) [10]. The substances were administered by injection into the neck muscles of the animals in the postoperative period.

**Table 1:** Day of injection and postoperative period for the fluorochromes

Postoperative time	Tetracykline	Xylenol orange	Calcein green
4 weeks	Day 9	Day 16	Day 24
8 weeks	Day 37	Day 44	Day 52
12 weeks	Day 65	Day 72	Day 80

### Implant separation and preparation

The animals were killed in deep sedation after premedication at the scheduled postoperative time of 4, 8, or 12 weeks, by injection of 20–40 ml KCL 7.45% under strict ECG control. The death was ascertained by asystole, auscultation, and a missing corneal reflex. The procedure is described by Seidling et al. [10]. Shortly summarized, the preparation of the undecalcified samples was performed according to Donath and Breuner which is a method for embedding and processing of jaw bones with teeth or bone with ceramic and metal implants [31]. A modified Masson-Goldner staining was used to differentiate between mature bone and immature osteoid [32]. The sample selection and preparation procedure has already been described by Seidling et al. [10] and Lehmann et al. [33] previously. In particular, the very middle specimen of the serial cuts was used.

### Evaluation

The histomorphometric evaluation was carried out interactively using a Leica DRC 300 FX-microscope (Fa. Leica Camera AG, Solms, Germany). For this study, a specific evaluation program (“Macro”) has been created with the Leica company based on the Leica Qwin V3 software (Leica Camera AG, Solms, Germany) [10]. This allows a computer-based assessment on the basis of mosaic images of the complete gap under higher magnification. The samples were sighted on the microscope while using the semiautomatic evaluation program and marking the different types of bony tissues namely osteoid and bone respectively. The created Macro has been validated previously [34]. The variables of bone and osteoid ongrowth as indicators for the osseoconduction, as well as bone and osteoid volume as indicators for the osseoinduction, were able to be determined due to the use of the staining according to Masson-Goldner [10, 32].

All slides were checked and then blinded prior to the histomorphometrical evaluation by three independent investigators (ML, RS, EM). A ROI of 5 mm width in the middle of the gap was used according to the prior investigation. All non-evaluable preparations were sorted out. As exclusion criteria, e.g., a partial gap coverage and surface detachment of the TPS surface layer were identified [10]. The evaluators repeated measurements on a specific specimen for self-supervision [10].

For the microscopic evaluation with a Leica DRC 300 FX (Leica Camera AG, Solms/Germany; used filters: LeicaI3 = DAPI/Leica N2.1 = Rhodamin/Leica A = FITC) of the polyfluorochrome labelling, semi-quantitative scores were used, which are represented by means of a subjective assessment of the activity in general as an overview (general score), the amount of the observed fluorescence quantitatively for the de novo bone formation (amount score), and the intensity of the observed fluorescence for the bone turnover (intensity score) [10]. Table 2 shows the scores used and their assessment.

**Table 2:** Scores for the polyfluorochrome labeling

Points	General score	Amount score	Intensity score
0	None	No bone	None
1	Uncertain	Minor	Very low intensity
2	Minor	Moderate	Minor
3	Much	Much	Moderate
4	X	X	Much
5	X	X	Very much

### Statistics

The data was prepared in cooperation with the Department for Medical statistics, Biomathematics, and Information Processing, Medical Faculty Mannheim of the University of Heidelberg.

The four relevant outcomes of this trial were osteoid ongrowth, bone ongrowth, bone volume, and osteoid volume. Due to the non-normal distribution of the preparations (Kolmogorov test) and missing of homoscedasticity in a Levene’s test, the data experienced a logarythmization after addition of 1 (bone volume and osteoid volume) or 10 (bone ongrowth and osteoid ongrowth). After this transformation, data revealed to be approximately normally distributed with similar variances in the subgroups.

With these data, analysis of variance (ANOVA) with repeated measurement design was performed using SAS release 9.4 (SAS Institute, Cary / North Carolina, USA). The SAS procedure “PROC MIXED” has been applied for two- or three-way ANOVAS where the parameters group (“type of implant”), localization (hip or knee), and postoperative time (4, 8, and 12 weeks) have been considered as fixed factors and animal ID as a random factor. We used these techniques in order to analyze three factors simultaneously and to test for interactions. If necessary, pairwise post hoc comparisons have been done using Tukey’s test.

For the statistical evaluation of the intravital staining, only a descriptive statistics was carried out using Excel for Mac Version 2011 (Microsoft Company, Washington/USA). The level of significance was *α* = 0.05. A sample size calculation was performed as described by Seidling et al., previously [10].

## Results

### Amount of implants

In total, 95 implants of 108 were able to be evaluated. At least 9 implants of 12 were able to be assessed in one CSA group and one BMP-2 group. Implants were lost in several groups due to the defined exclusion criteria.

The following line-up of the three-way ANOVA and the pairwise comparison between the groups BMP-2, TPS, and CSA give a closer look at the results.

### BMP-2 vs. TPS

The three-way ANOVA showed significant differences for the variables bone ongrowth (implant type), osteoid volume (implant type), and osteoid ongrowth (implant type and postop time) in the pairwise comparison between TPS and BMP-2 (Table 3).

**Table 3:** The calculated *p* values of the three-way ANOVA of the comparison BMP-2 vs. TPS using the “PROC MIXED” of SAS 9.4 (level of significance is 0.05; “*” shows significant values)

	Bone volume	Osteoid volume	Bone ongrowth	Osteoid ongrowth
Implant type	0.2143	0.0324*	0.0012*	< 0.0001*
Postoperative time	0.8643	0.4614	0.1823	0.0352*
Location	0.0682	0.1151	0.1050	0.9518
Implant type x postoperative time	0.3327	0.5861	0.3799	0.2299
Implant type x location	0.1945	0.7746	0.2297	0.2944

**Fig. 1:**
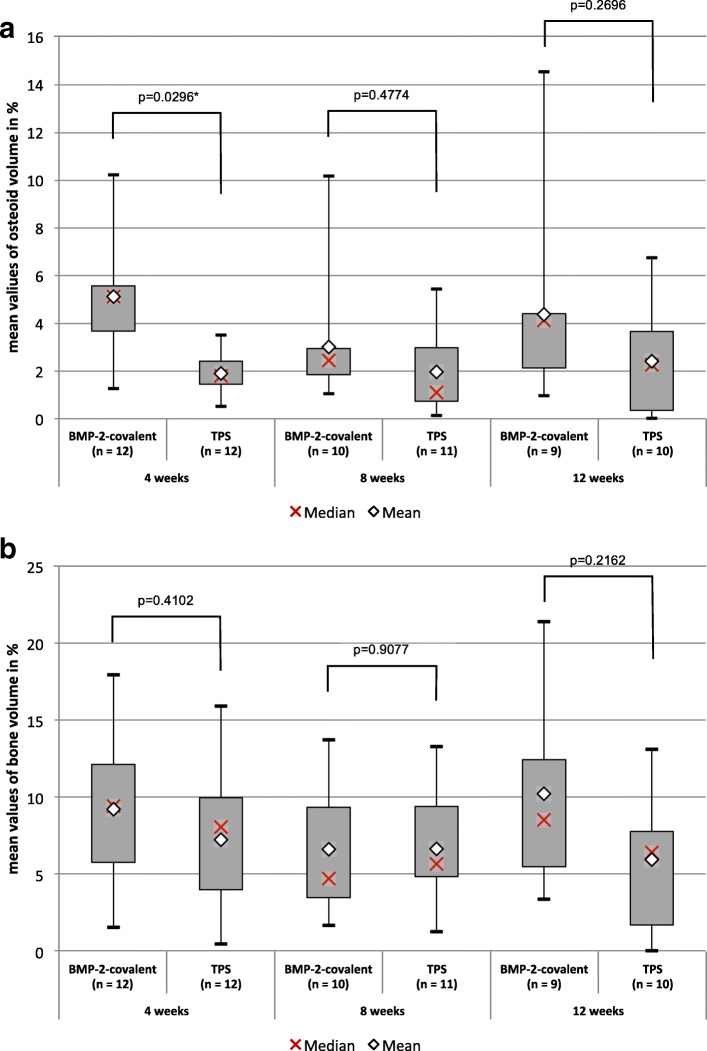
Comparison of the BMP-2 group and the TPS group for osseoinduction. **a** Osteoid volume and **b** bone volume by group (BMP-2 vs. TPS) and postoperative duration (4, 8, and 12 weeks). Only for the amount of osteoid in the gap, a significant difference was able to be shown

After a healing period of 4 weeks, there was an increased amount of osteoid in the gap, shown by the variable of osteoid volume with mean values of 5.13% (SD ± 2.58%) for covalently bound BMP-2 vs. 1.89% (SD ± 0.85%) for the TPS layer. The remaining values of osseoinduction showed post hoc no significant difference (Fig. 1).

**Fig. 2:**
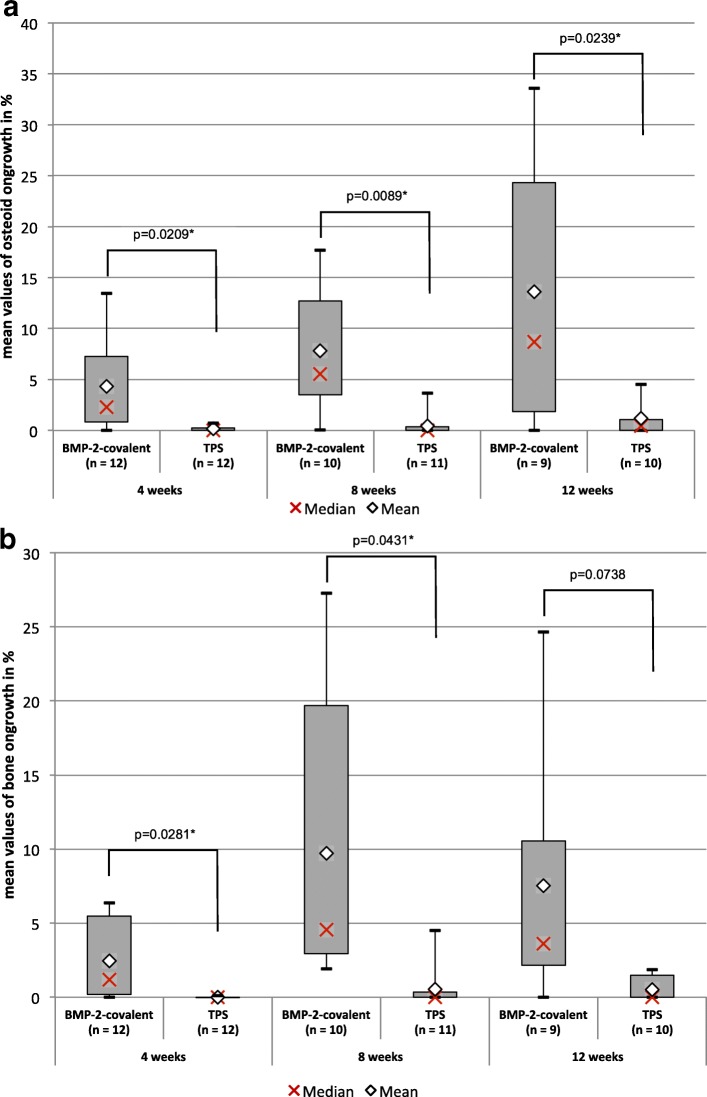
Comparison of the BMP-2 group and the TPS group for osseoconduction. **a** Osteoid ongrowth and **b** bone ongrowth by group (BMP-2 vs. TPS) and postoperative duration (4, 8, and 12 weeks). For the osteoid to implant contact, expressed by the variable osteoid ongrowth, a significant difference between BMP-2 and TPS over the whole observation period was able to be shown, in favor of BMP-2. After 4 and 8 weeks, there was significantly more bone to implant contact (bone ongrowth) in the BMP-2 group with no statistically significant difference after 12 weeks

After 4 weeks, there was significantly more bone to implant contact, expressed by the variable bone ongrowth, with a *p* value of 0.0281 (BMP-2 2.47% ± 2.63% vs. TPS 0.01% ± 0.03%). Also after 8 weeks, we observed an increased bone to implant contact with a *p* value of 0.0431 (BMP-2 9.71% ± 9.25% vs. TPS 0.53% ± 1.34%) (Fig. 2). After a healing period of 12 weeks, there was no significant difference (Fig. 2).

For the osteoid to implant contact, we observed a statistically significant increase over the whole observation period (Fig. 2). After a healing period of 4 weeks, we found an increased osteoid ongrowth with amounts of 4.32% (SD ± 4.34%) for BMP-2 vs. 0.15% (SD ± 0.26%) for TPS. After a healing period of 8 weeks, we observed an increased contact of osteoid to the implant with amounts of 7.81% (SD ± 5.82%) for BMP-2 vs. 0,46% (SD ± 1.08%) for TPS. We also observed an increased amount after a period of 12 weeks with 13.59% (SD ± 13.44%) for BMP-2 and 1.18% (SD ± 1.74%) for TPS (Fig. 2).

### CSA vs. BMP-2

The three-way ANOVA showed significant differences for the variables bone volume (location) and osteoid ongrowth (postoperative time) in the pairwise comparison between CSA and BMP-2 as shown in Table 4. The post hoc test for the interaction between implant type and postoperative period give a closer look on the osseointegration.

**Table 4:** The calculated *p* values of the three-way ANOVA of the comparison BMP-2 vs. CSA using the “PROC MIXED” of SAS 9.4 (level of significance is 0.05; “*” shows significant values)

	Bone volume	Osteoid volume	Bone ongrowth	Osteoid ongrowth
Implant type	0.9213	0.7129	0.3260	0.2602
Postoperative time	0.5053	0.1498	0.0627	0.0081*
Location	< 0.0001*	0.1140	0.0927	0.9597
Implant type x postoperative time	0.4037	0.3262	0.5878	0.7636
Implant type x location	0.2097	0.7716	0.5963	0.3977

**Fig. 3:**
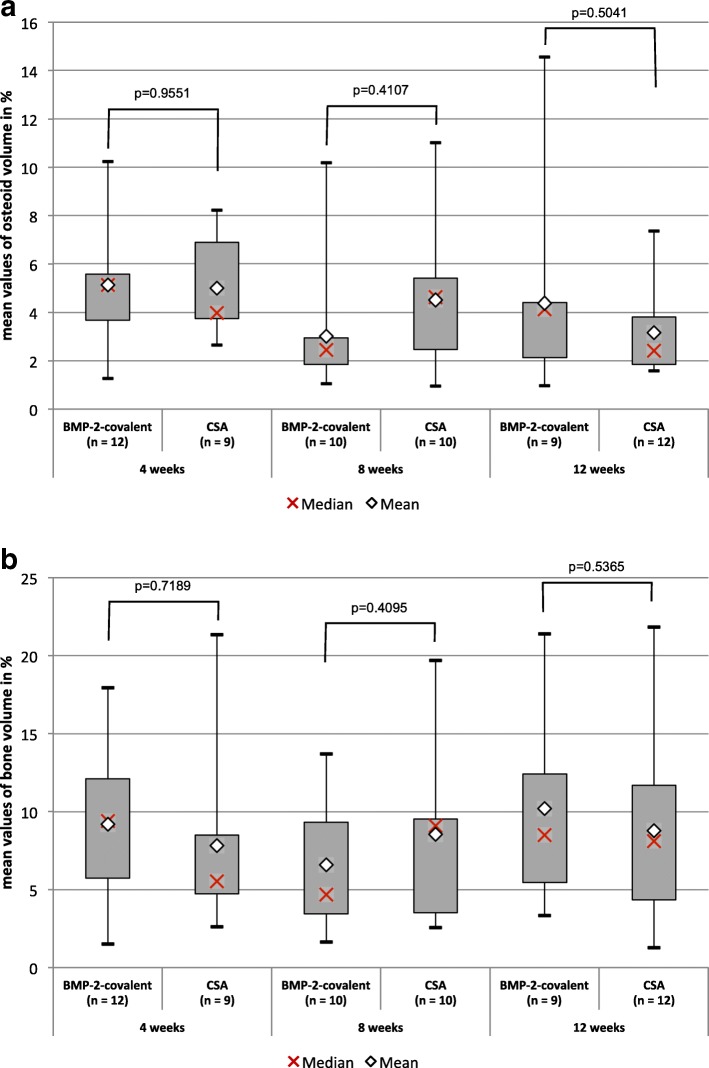
Comparison of the BMP-2 group and the CSA group for osseoinduction. **a** Osteoid volume and **b** bone volume by group (BMP-2 vs. CSA) and postoperative duration (4, 8, and 12 weeks). No statistically significant difference was able to be shown in the pairwise comparison of BMP-2 and CSA for the variables bone and osteoid volume

For the osseoinduction, expressed by the variables bone and osteoid volume, in the pairwise comparison between the CSA group and the BMP-2 group, there was no statistically significant difference seen over the complete observation period of until 12 weeks as shown in Fig. 3.

**Fig. 4:**
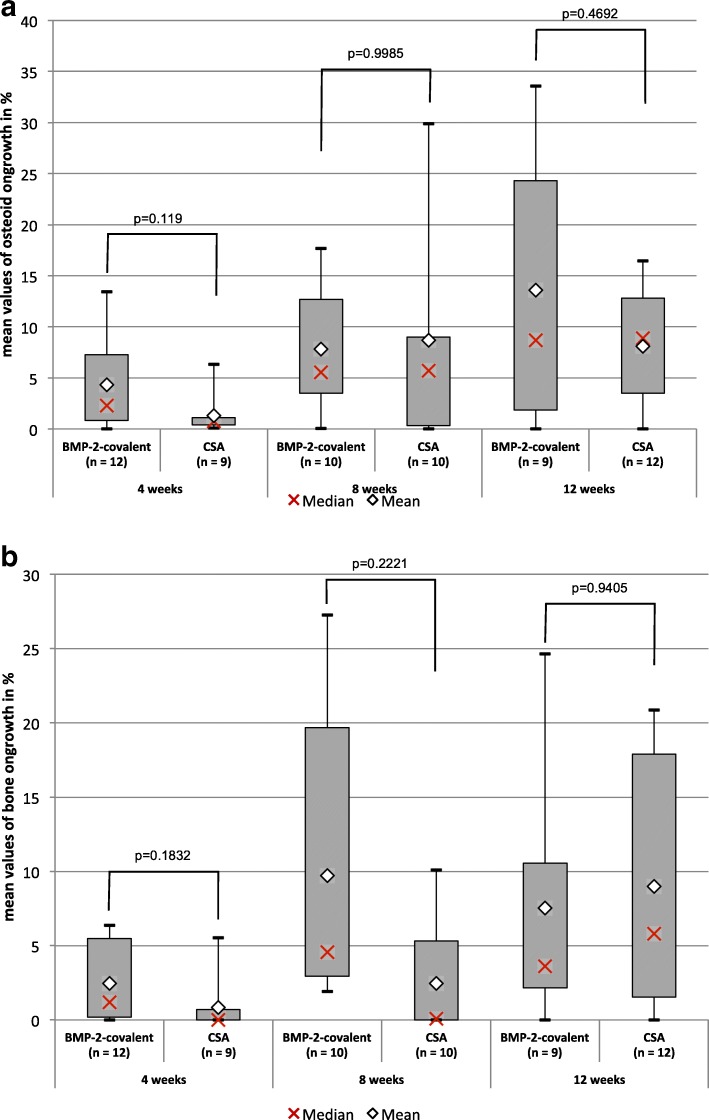
Comparison of the BMP-2 group and the CSA group for osseoconduction. **a** Osteoid ongrowth and **b** bone ongrowth by group (BMP-2 vs. CSA) and postoperative duration (4, 8, and 12 weeks). There was no statistically significant difference between the groups BMP-2 and CSS to be shown for the bone or osteoid-to-implant contact by the pairwise comparison

There was 1.7 to threefold more osteoid in contact to the implant in favor of BMP-2. No statistically significant differences could be shown after the 4, 8, and 12 weeks of healing period. Also, there was a 2.9 to 3.9-fold more bone to implant contact in the BMP-2 group, but there was also found no significant difference after a healing period of 4, 8, and 12 weeks. In a nutshell, there was no significant difference found between the results of the CSA and the BMP-2 group (Fig. 4).

### Implant location

In the contemplation of the implantation site (hip vs. knee) within the BMP-2 group over the postoperative periods of 4, 8, and 12 weeks, there was additionally a significant difference found for the variable bone volume with a *p* value of 0.0496 in favor of the hip localization with mean values of 6.87% (SD ± 4.94%) for the location knee and 10,54% (SD ± 4.91%) for the hip.

### Intravital staining

**Fig. 5:**
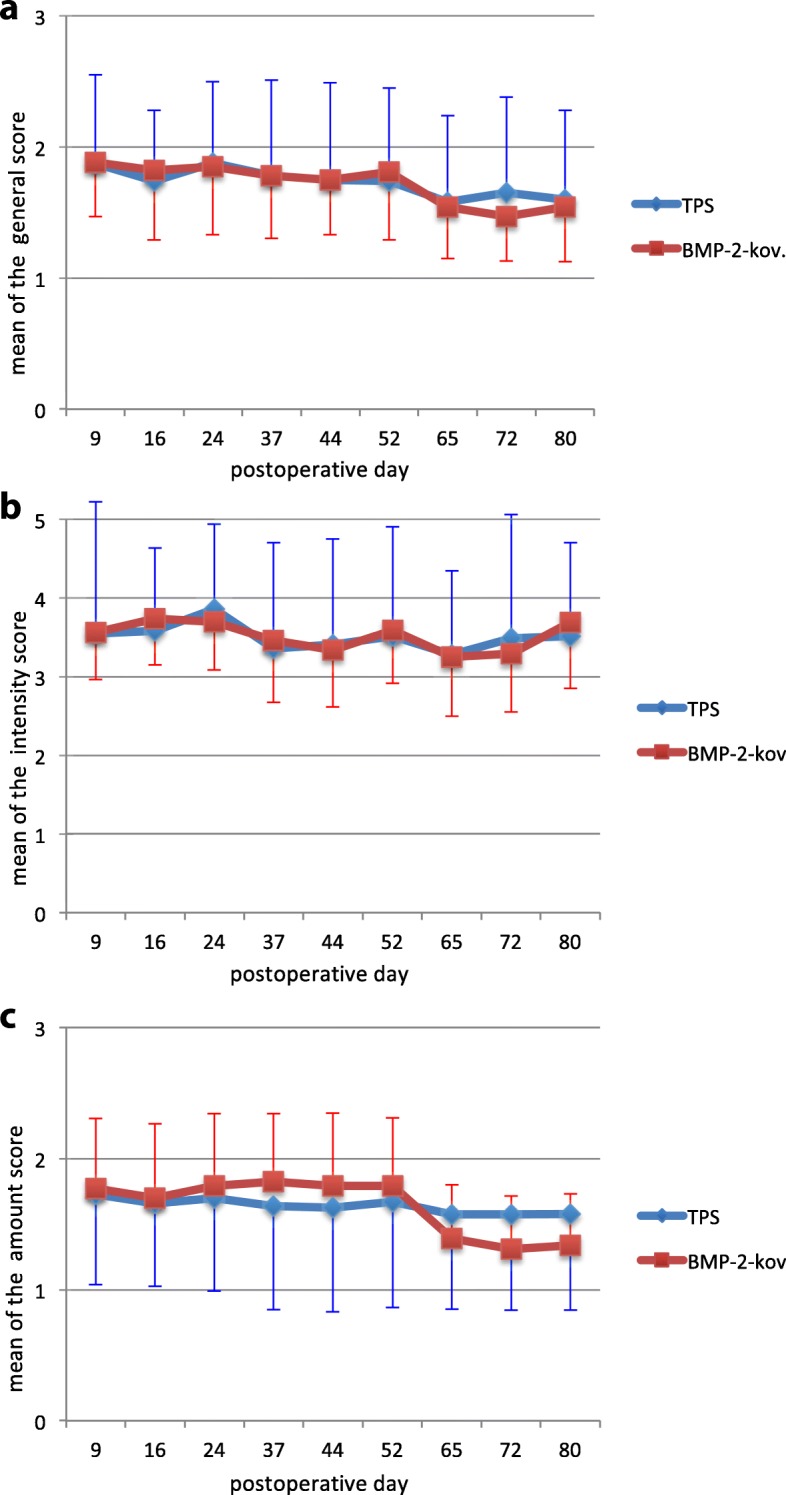
Intravital staining by postoperative period for the **a** general score, **b** intensity score, and the **c** amount score. The error indicators correspond to the standard deviations

We observed bone turnover over the whole postoperative period of 12 weeks. There was an inconsistently but slight bone turnover, dependent on the used score (Fig. 5). Depending on the score used, we found relatively small differences between TPS and BMP-2 to the max. 17% (quantity score), with relatively large variances (up to 49% of the mean value).

## Discussion

The aim of the study was to evaluate the osseointegrative force of rhBMP-2 in a large animal.

We have seen the main effect in the formation of osteoid and bone at the surface of the implant less inside the gap. And the effect of the BMP-2 protein seems to be pronounced more for the growth of osteoid as a precursor of bone than at mature bone. Seen from a temporal perspective, one can assume that the appearance of osteoid is close to the recruitment of preosteoblasts. In the present study, we found an indication of new osteoid formation at the beginning after 4 weeks which we interpret as osteoinduction. This can be correlated to the explanations of Albrektsson and Johannson [35]. They point out that the osteoinduction may start immediately after injury (in the present study the implantation) with a high activity over the first weeks as the recruitment of preosteoblasts may not be evident until some weeks later. This may explain also our results that we did not see any further effects in the gap in terms of osteoinduction later on.

Several chemotactic properties have been demonstrated for BMP-2 which is discussed as responsible for attracting corresponding effector cells [36–39]. Presumably, this property contributes to the influence of osteoconduction after long-term fixation of the BMP-2 to surfaces [5]. Since the significant differences were found mainly in the immature bone-like osteoid volume and osteoid ongrowth in the present study, it can be assumed that the effects of BMP-2 have not yet been completely assessed as the observation time was limited to 12 weeks. Further investigations should be performed to show effects at a later time.

The used BMP-2 coating was covalently bound to the titanium TPS surface by prior treatment with chromosulfuric acid and binding via anchoring molecules and maintains a contact angle of 0–8° for 50 days [25]. The biological activity is reported as still evident [25] shown in cell culture by detection of alkaline phosphatase as an indicator of bone induction by BMP-2. Thus, the results of the present study can close the gap between in vitro and in vivo assessment together with preliminary studies with the same bioactive layer [40]. Sumner and coworkers made a study to analyze the influence of different dosage of BMP-2 on top of a HA/TCP. They found a 2–3.5-fold increase of bone formation in the gap which was 3 mm in height [13]. They compared a previously reported group with HA/TCP to recent groups with locally delivered rhBMP-2 in the left humeri and implants with solely buffer in the right humeri. Effects on the contralateral site were observed with an enhanced bone ingrowth. So, Sumner and colleagues hypothesized that not the BMP-2 but rather factors released by the regeneration process are responsible for this reported contralateral effect. Thus, a systemic effect could be discussed using BMP-2 as implant coating. In the present study, the control groups were separate pigs with only BMP-2 or CSA modification or none (TPS). Thus, an effect on control implants which were set in the same animal was excluded by the study design in the present trial.

As previously described, the implanted specimens were subjected to a treatment with chromosulfuric acid at high temperatures prior to biocoating with BMP-2 via anchor molecules, a process known as “surface enhancement” [41]. It has already been shown that this has a positive effect on osseoinduction as well as on osseoconduction [10, 40]. This effect, however, appears to be higher after addition of BMP-2. But, the present results show markable differences between BMP-2 covalent and CSA, which are all not significant post hoc. Considering the results of the paired comparison of CSA and TPS in particular as described by Seidling and coworkers [10], significant differences were seen for the variables osteoid ongrowth and (12 weeks) osteoid volume (4 weeks). Thus, the effect does not appear to be due solely to the surface modification.

The used gap model allows the differentiation between the osteoinductive and the osteoconductive properties of a layer on an implant in the bone [10]. Gap models are known as models for healing and turnover research in bone [14, 42, 43]. It is also known as a model to examine some sealing effects between the implant and bone [12] when a gap results between the surface of an implant and the bone stock which should be filled by bone. A sealing effect can also be an important feature of a BMP-2 coating in primary treatment with artificial joints or even with revision surgery when a loss of the bone stock came up and gaps between implant and bone are likely [10, 44, 45]. At the other hand, a gap could be seen as a carrier system like a potential reservoir between implant and bone for BMP enhancing osteogenesis, e.g., for the treatment of nonunions after bone fractures by local delivery of the protein [46, 47]. We additionally observed a significant difference for the implant site (hip vs. knee) and the variable bone volume in the three-way ANOVA. To what extent, there is an interaction between the site of implantation (knee or hip) and the surface could only be observed. Since it was not content of the present study, further observations are needed to clarify this observation.

Proteins need appropriate carrier systems when bound at the surface of an implant to keep their biological activity [3, 7, 41, 45, 48]. Several bonding techniques are described [49]. The specific technique for bonding the BMP-2 at the TPS surface of the used implants is described by Jennissen and his group [3, 41] and was performed in the present study.

Beneath the choice of the appropriate time point the gap model may have disadvantages for the analysis of the osteoinduction properties of the BMP-2 coating of implants. The BMPs were detected when the bone was implanted into the tissue like the musculus rectus abdominis or quadriceps or in bone [50]. Looking at the situation in the gap model where alloplastic material is used at the implant site blood supply has to be missed in consequence.

A perfect animal for all types of bone research may not exist as there is a high variation in, e.g., bone turnover [51]. Especially in small animals, the bone turnover seems to be much higher than in humans or large animals [51]. As previously described, the lamellar bone structure of Göttingen minipigs is nearly similar to humans, as well as the remodeling sequences [17, 52]. Furthermore, the bone marrow and marrow stem cells of Göttingen minipigs have similar characteristics to humans, especially in proliferation and morphology [16]. Not least, there is a high experience with this type of animals in orthopedic research, and they are cost-effective and easy to handle [53]. Nevertheless, none of the common study animals like dogs, pigs, or rats is fully comparable with humans [52].

The analysis of the intravital staining revealed bone turnover over the whole observation time. None of the used scores was able to distinguish between the BMP-2 and the TPS group clearly as the values laid close together combined with wide standard deviations for both groups. The wide variances may also be caused by the differing results of the examinations of the three observers (data not shown) reflecting the individual character of the evaluation. However, the activity of bone turnover was not constant in the scores over the observation time. The “general score” and the “amount score” seemed to diverge at the end of the observation time, whereas the “intensity score” shows an undulating behavior.

However, the semi-quantitative nature of the assessment must be considered. In particular, the used scores are highly dependent on the investigator. Furthermore, the light-emitting behavior of the substance in the mineralization front is quite different and depends above all on the processing and handling of the preparations [20]. Pautke et al. [20] observed no bleaching effect for the substances xylenol orange, calcein green, and rolitetracycline during storage in the dark and room temperature over a period of 6 months. Only the substance calcein blue showed a disappearance of the fluorochrome effect after 6 months [20]. Due to the experimental setup, the temporal distance between implantation and evaluation was more than 6 months; so, a bleaching cannot be safely excluded. Comparing both techniques, the histomorphometry and the intravital staining, we guess that the histomorphometry is more effective than the intravital staining in the assessment of osseointegration of implants, not least because of the low intra- and interobserver reliability.

In general, some drawbacks of BMP-2 have to be considered when used. According to the basic characteristic of the protein, ectopic bone formation may be possible after implantation in some case reports we have found in literature [54–57]. However, the working group of the present study was able to exclude a side effect in this case in terms of new bone formation at another site of the treated animals [33].

A review, published by Carragee et al. [11], brought BMP-2 into disrepute in the application to humans. Carragee et al. described a clear study design bias with an actually 10–50-fold increased complication rate compared to the previously published data [11]. There was a 40% increased risk of adverse events compared to the conventional autologous bone graft, as well as an increased risk of malignant disease in the absence of benefit of the BMP-2 [11, 58, 59]. Since rhBMP-2 is a derivative of a differentiation factor in the mesenchymal development, malignant degeneration is not unlikely [54, 60]. A participation in tumor angiogenesis, as well as an inhibition of tumor cell apoptosis, could also be demonstrated [39].

Thus, the harmlessness of BMP-2 should be shown in further studies as this question was not the rationale of the present study dealing with the osseointegration.

## Conclusion

Covalently bound BMP-2, applied to a titanium sample body after prior surface modification with chromosulfuric acid, had positive effects on the osseointegration in the large animal model during the observation period of 12 weeks. The osseoconduction, expressed by the variables osteoid ongrowth and bone ongrowth, was mainly influenced. Due to the lack of significant differences in the pairwise group comparison between BMP-2 and CSA, further studies should show a benefit of the BMP-2 against the sole surface modification even more clearly. The results showed that the effect is probably not explainable by the treatment with chromosulfuric acid solely rather by an additional application of BMP-2.

## Abbreviations

BMP-2: Bone morphogenetic protein-2
CSA: Chromosulfuric acid (acronym for the CSA surface)
GMP: Göttingen minipig
ROI: Region of interest
SD: Standard deviation
TPS: Titan plasma spray (acronym for the TPS surface)
